# Hyaluronan-Based Hydrogel Hybrid Insulin Carriers—Preformulation Studies

**DOI:** 10.3390/polym17192661

**Published:** 2025-10-01

**Authors:** Aneta Ostróżka-Cieślik

**Affiliations:** Department of Pharmaceutical Technology, Faculty of Pharmaceutical Sciences in Sosnowiec, Medical University of Silesia, Jedności Street 8b, 41-200 Sosnowiec, Poland; aostrozka@sum.edu.pl

**Keywords:** hydrogels, insulin, alginate, hyaluronan, hydroxypropyl methylcellulose, rheology, texture, Strat-M^®^ membrana

## Abstract

This paper proposes hybrid hydrogel insulin carriers based on alginate-hyaluronan (ALG/HA) and hydroxypropyl methylcellulose-hyaluronan (HPMC/HA) for topical application. The inclusion of insulin in a modern dressing can help restore metabolic balance and proper cell signaling in diseased tissue. Preformulation studies of the developed preparations were conducted, including analysis of the in vitro pharmaceutical availability of insulin, rotational and oscillatory rheology tests, and texture profile analysis. It was found that the developed insulin formulations provide an acceptable compromise between rheological and textural properties and ease of application, while ensuring prolonged release of the active substance. The results obtained provide a basis for further preclinical and clinical studies.

## 1. Introduction

The treatment of chronic wounds is a major problem for modern medicine and an economic challenge in healthcare. It is estimated that this condition affects approximately 1.5% of the population and is steadily increasing. A key element of the treatment process is the use of specialised therapies and preparations with antiseptic, anti-inflammatory, regenerative, and moisturising properties. Chronic wounds with exudate, which are susceptible to bacterial colonisation, are particularly difficult to treat [1]. Hydrogels are highly effective in treating this type of wound. According to the definition of the European Pharmacopoeia, it is a semi-solid form of a medicinal product consisting of a mixture of water with glycerol or polyglycol, thickened (gelled) with polymers [2].

It is suggested that hydrogels based on hyaluronan, alginate, and hydroxypropyl methylcellulose are effective in the treatment of chronic wounds. It has been found that the use of an alginate dressing shortens wound healing time in a mouse model to 10 days compared to a normal wound healing for 2–3 weeks [3]. Studies conducted in a rat excised wound model confirmed the therapeutic efficacy of a hydrogel combining alginate (ALG) and hyaluronan. Wound closure occurred within 5 days (compared to ALG *p* < 0.001) [4]. Alginates are natural copolymers composed of β-D-mannuronic and α-L-guluronic acids, linked by [1,4] glycosidic bonds [5]. They are widely used in regenerative medicine [6]. They have the ability to absorb exudate at the wound site and maintain a moist microenvironment, which promotes healing and granulation tissue formation [4]. Hyaluronan consists of units of (β,1-4)-d-glucuronic acid and (β,1-3)-N-acetyl-d-glucosamine. In its high-molecular-weight form (>100 kDa), HA occurs naturally in tissues, including the dermis and epidermis [7]. It has been found to be a regulator of tissue hydrodynamics. It participates in tissue repair, modulates wound inflammation, and increases keratinocyte migration and proliferation [8,9]. The effectiveness of HA in the healing of experimentally induced wounds in rats and hamsters and in the treatment of diabetic foot ulcers has been confirmed [9,10]. A review of the literature indicates a positive effect of cellulose derivatives on the wound healing process. They can be used as a standalone drug carrier or in combination with natural, semi-synthetic, or synthetic polymers. They have been found to improve the physicochemical properties of dressings and have a beneficial effect on diseased tissue. Hydroxypropyl methylcellulose is a cellulose ether used as a hydrophilic hydrogel API carrier in various concentrations. It is non-toxic, exhibits bioadhesive properties, and increases viscosity [11,12].

Hydrogels based on alginate, hyaluronan, and hydroxypropyl methylcellulose may be potential carriers of biomolecules, including insulin. This hormone has been confirmed to be a powerful stimulator of wound healing, as evidenced by numerous preclinical and clinical studies [13,14,15,16,17,18,19,20]. It has been found that incorporating insulin into a modern dressing can restore metabolic balance and normal cell signaling in diseased tissue. The importance of insulin incorporated into a hydrogel matrix in wound treatment has been discussed in detail in previous articles co-authored by me [13,14,15]. It is suggested that this is an effective and safe method for accelerating the healing of chronic wounds [13,14,15,21].

The aim of the study was to develop a hybrid hydrogel insulin (INS) carrier in an alginate-hyaluronan (ALG/HA) and hydroxypropyl methylcellulose-hyaluronan (HPMC/HA) system for topical application.

## 2. Materials and Methods

### 2.1. Materials

Insulin Insulatard Penfil (INS, 100 IU/mL, suspension of human insulin, isophane, long-acting) was supplied by Novo Nordisk (Bagsværd, Denmark). The preparation contained the following excipients: zinc chloride, glycerol, protamine sulphate, sodium hydroxide, disodium phosphate dihydrate, metacresol, phenol, hydrochloric acid, and water for injections. Hydroxypropyl methylcellulose was sourced from Sigma Chemical Co., St. Louis, MO, USA. PBS (buffered phosphate saline solution; pH = 7.4) was sourced from Sigma-Aldrich, St. Louis, MO, USA. Sodium hyaluronate (molecular weight 1.5 MDa) was obtained from Chemat, Gdańsk, Poland. Sodium alginate was purchased from Agnex Sp. z o. o., Białystok, Poland. Calcium chloride dihydrate was purchased from POCH S.A., Gliwice, Poland. Glycerol (86%) was supplied by PPH Microfarm, Zabierzów, Poland. The substances used were of p.a. purity. The Strat-M^®^ membrane was sourced from Merck Millipore (Burlington, MA, USA).

### 2.2. Methods

#### 2.2.1. Preparation of Hybrid Hydrogels

##### Preparation of a Hydrogel Matrix Based on Hydroxypropyl Methylcellulose and Sodium Hyaluronate (HPMC/HA)

The hydroxypropyl methylcellulose (HPMC) hydrogel was prepared by dissolving 4.0% HPMC in a mixture of 93.0% PBS and 3.0% glycerol, previously heated to 80 °C. The sodium hyaluronate (HA) hydrogel was prepared by dissolving 0.5% HA in 99.5% PBS. The resulting formulations were combined in a 2:1 ratio (HPMC:HA) to obtain a homogeneous hybrid hydrogel. The individual components and the final formulation were prepared using a stirrer equipped with a Fisherbrand Isotemp heating plate (Thermo Fisher Scientific, Mississauga, ON, Canada). The rotation speed was set at 1000 rpm. The preparation was left to cross-link for 7 days at 2–8 °C, after which insulin was mechanically introduced (1 mL per 2.5 g of substrate/28.57 IU/g). The hydrogel matrix (HPMC/HA) was stored between tests in a refrigerator at 4 °C. The hydrogel had a pH of 7.45. The analysis was performed using a SevenCompactTM S210 laboratory pH meter equipped with an InLaB^®^Expert Pro-ISM electrode (Mettler-Toledo GmbH, Greifensee, Switzerland). The osmotic pressure of the preparation was 448 mOsm/L (Gonotec Osmomat 3000, Gonotec GmbH, Berlin, Germany). The mechanical stability of the hybrid hydrogel was tested using a centrifugation method. 1 g of the sample was placed in an Eppendorf tube and centrifuged at 4000 rpm for 30 min in a microcentrifuge (Model MPW-300, MPW Med. Instruments, Warsaw, Poland).

##### Preparation of a Hydrogel Matrix Based on Sodium Alginate and Sodium Hyaluronate (ALG/HA)

5.0% sodium alginate was dissolved in a mixture of 83.0% PBS and 10.0% glycerol. Then, 1.0 g of 0.5% calcium chloride (CaCl_2_) was added, stirring until the hydrogel cross-linked. The formulation containing sodium hyaluronate (HA) was prepared by dissolving 0.5% HA in 99.5% PBS. The resulting components were combined in a 1:1 ratio (ALG:HA) to obtain a homogeneous hybrid hydrogel. The individual formulations and the final hydrogel matrix were prepared using a stirrer equipped with a Fisherbrand Isotemp heating plate (Thermo Fisher Scientific, Mississauga, ON, Canada). The rotation speed was set at 1000 rpm. The preparation was left to cross-link for 7 days at 2–8 °C, after which insulin was mechanically introduced (1 mL per 2.5 g of substrate/28.57 IU/g). The hydrogel matrix (ALG/HA) was stored between tests in a refrigerator at 4 °C. The hydrogel had a pH of 7.42. The analysis was performed using a SevenCompactTM S210 laboratory pH meter equipped with an InLaB^®^Expert Pro-ISM electrode (Mettler-Toledo GmbH, Greifensee, Switzerland). The osmotic pressure of the preparation was 974 mOsm/L (Gonotec Osmomat 3000, Gonotec GmbH, Berlin, Germany). The mechanical stability of the hybrid hydrogel was tested using a centrifugation method. 1 g of the sample was placed in an Eppendorf tube and centrifuged at 4000 rpm for 30 min in a microcentrifuge (Model MPW-300, MPW Med. Instruments, Warsaw, Poland).

#### 2.2.2. Study of the Pharmaceutical Availability of Insulin from Hydrogels in Vitro

The analysis of pharmaceutical availability was performed using an Erweka DT600 paddle apparatus (Husenstamm, Germany) equipped with a Dissolution Enhancer Cell™ (exposure area of 3.80 cm^2^; Erweka, Husenstamm, Germany). A sample of hydrogel with insulin (1 g) was placed in the diffusion chambers (*n* = 6), and a Strat-M^®^ membrane (imitating the skin barrier) was applied in accordance with the manufacturer’s instructions. The chamber was placed at the bottom of the vessel (volume 200 mL). The release process parameters were set as follows: PBS acceptor fluid volume: 50 mL; temperature: 32 °C ± 1 °C (temperature on the surface of human skin); mini-paddle rotation speed: 100 rpm. The amount of insulin released was measured using a Cecil UV-VIS spectrophotometer (CE 3021, Cambridge, UK). The analytical wavelength for spectrophotometric measurements (λ = 271 nm) [22,23]. A linear relationship between absorbance and the concentration of standard solutions was obtained, described by the equation y = 0.453x + 0.0072 (R^2^ = 0.999). The precision of the method was assessed positively based on the values of standard deviation, relative standard deviation, and coefficient of variation of the method.

The obtained insulin release profiles from hydrogels were evaluated in terms of similarity. Statistical methods of similarity (f2) and difference (f1) factors were used, which are recommended by the US Food and Drug Administration (FDA) and the European Medicines Agency (EMA) [24]. DDSolver 1.0 software (add-in for Microsoft Excel 2019) [25] was used for the analysis.

DDSolver 1.0 software (add-in for Microsoft Excel 2019) [25] was used to analyse the kinetics of insulin release from hydrogels. Mathematical models were used to describe the API release mechanism: zero-order model, first-order model, Higuchi model, Korsmeyer–Peppas model, Hixson–Crowell model, Peppas–Sahlin model, and Weibull model. The following were used to assess the compliance of the models with the obtained data: the coefficient of determination R^2^ (a higher R^2^ value indicates a better fit to the model), the Akaike information criterion (a lower AIC value indicates a better fit to the model) and the model selection criteria (the highest MSC value indicates a better fit to the model).

#### 2.2.3. Rheological Analysis

##### Rotational Test Measurements

The rheological properties of hydrogels were tested using an RM 200 rotational rheometer (Lamy Rheology Instruments, Champagne au Mont d’Or, France). Rheological characteristics were determined at a temperature of 25 °C ± 0.1 °C (storage temperature and temperature of insulin hydrogel taken from the unit packaging) and at 32 °C ± 0.1 °C (skin surface temperature), using the MK-CP 2445 measurement system in a plate/plate geometry (diameter of 24 mm, angle: 0.45°). The measurement accuracy was ±1%, while the repeatability was ±0.2%. A constant temperature during the measurements was maintained using the Lamy Rheology CP-1 PLUS heating system. A sample with a volume of approximately 1 mL was applied to the measuring element and left for 30 min to reach equilibrium. It was then subjected to variable shear rates in the range of 7.0 s^−1^ to 100.0 s^−1^. The measurement time was 15 min. Thixotropy analysis was performed using the hysteresis loop method. Data analysis was performed using Rheomatic-P software (Version: 2.1.0.4). The flow curves were analysed based on selected mathematical rheological models: Casson, Bingham, Herschel–Bulkley, Ostwald–de Waele [26,27]. The degree of model conformity with the data was assessed using R^2^ (coefficient of determination), based on the assumption that the higher its value, the better the fit to the model.

##### Oscillatory Test Measurements

The analysis was performed using a Modular Compact Rheometer MCR302e from Anton Paar GmbH (Graz, Austria) with a plate/plate geometry (PP50, diameter 50 mm). The gap between the parallel plates was set to 0.5 mm. Two oscillation tests were performed: an amplitude sweep test and a frequency sweep test. The amplitude sweep test was performed at a constant frequency of 1 Hz, using a strain amplitude of 0.1 to 100%. The frequency sweep test was performed by reducing the oscillation frequency from 10 to 0.1 Hz, with a deformation range of 1%. The tests were performed at a temperature of 25 °C ± 0.01 °C (storage and removal temperature from the unit packaging) and 32 °C ± 0.01 °C (human skin surface temperature). Temperature control was provided by a Peltier system. The rheological properties of hydrogels, such as G′ (storage/elastic modulus) and G″ (loss/viscous modulus), were determined. The analysis was performed using Rheo Compas software (Version: 1.31).

#### 2.2.4. Texture Analysis

The texture properties of the developed hydrogels were determined using a Texture Analyzer TX-700 (Lamy Rheology Instruments, Champagne au Mont d’Or, France), equipped with a hemispherical probe with a diameter of 8 mm. During the CRT (Direct Compression/Relaxation/Tension) test, one compression cycle was performed under the following conditions: compression speed: 1.0 mm/s, relaxation time (or time between cycles): 20 s, distance: 5.0 mm. During the TPA (Tension/Penetrometry) test, two compression cycles were performed under the following conditions: compression speed: 1.0 mm/s, distance: 5.0 mm, force to start: 0.05 N. Hardness is the maximum force measured during the first (Hardness 1) and second compression cycles (Hardness 2), which indicates the strength of the hydrogel. Adhesiveness is the force required to overcome the attractive forces between the probe surface and the sample. Cohesiveness is the work required to deform the hydrogel as the probe moves downwards. Elasticity is the ability of the hydrogel to deform under an applied load and recover its previous shape after the load is removed. Relaxation determines how the polymer relieves stress at a constant deformation [23,28,29,30,31]. The tests were conducted at a temperature of 25 °C ± 0.1 °C. The analysis was performed using RheoTex software for TX-700, version TX-UK01/2019. A theoretical introduction to texture analysis was presented in a previous article [27].

#### 2.2.5. Statistical Analysis

The tests were performed in at least three replicates. Mean values are given together with standard deviations. Statistical analysis was performed based on a two-sided Student’s *t*-test in Statistica 12.0 (Statsoft, Krakow, Poland). Statistica version 13.1 software (StatSoft, Krakow, Poland) was used for the calculations.

## 3. Results

The conducted research is a continuation of my previous analyses on the development of an effective insulin carrier to support the treatment of chronic wounds [23,26,27]. The research carried out so far confirms that hydrogels are an optimal API delivery system and an effective dressing material. They maintain a moist wound environment, provide a safe contact layer, have the ability to absorb and release the active substance, and are biocompatible and non-toxic. In addition, they absorb exudate and support tissue healing and regeneration processes [32].

Taking the above assumptions into account, hybrid insulin hydrogel matrices based on hydroxypropyl methylcellulose and sodium hyaluronate (HPMC/HA) and sodium alginate and sodium hyaluronate (ALG/HA) were developed. The HPMC/HA-INS hydrogel was translucent with a milky colour, while the ALG/HA-INS hydrogel was transparent. Both hydrogels were homogeneous and had a smooth texture. Their pH values were 7.45 and 7.42, respectively. It should be noted that the structure of hyaluronic acid is sensitive to acidity/alkalinity. At pH < 4 and pH > 11, it undergoes depolymerisation. This leads to the breakdown of hydrogen bonds, which play a key role in the structure of the HA molecule [33]. The pH value of the developed hydrogels will minimise the risk of irritation at the wound site. The developed hydrogels were hyperosmotic, with the HPMC/HA-INS hydrogel (448 mOsm/L) showing the value closest to physiological (300 mOsm/L). The hydrogels showed mechanical stability. No phase change or separation was observed.

**Figure 1:**
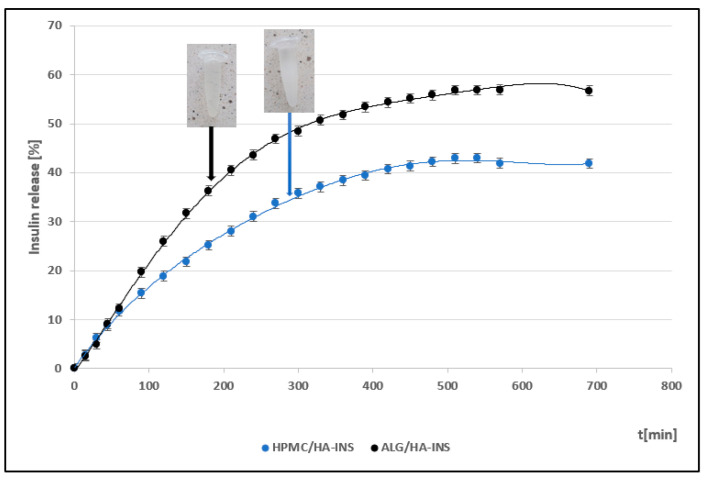
Insulin release profiles from prepared hydrogels (mean ± SD value (*n* = 6).

The pharmaceutical availability of insulin from the developed hydrogels was determined using a method based on API diffusion through a synthetic Strat-M^®^ membrane. The double-layer structure of this membrane (polyolefins and polysulfoethers) replicates the epidermis and dermis layers. It is suggested that the Strat-M^®^ membrane is the most optimal substitute for human skin for testing the release of active substances from epidermal drug forms [34]. The course of insulin release profiles from hybrid polymer matrices is shown in Figure 1.

By analysing the release profiles, it can be concluded that after 540 min, 43% and 57% of the initial API dose were released from the HPMC/HA-INS and ALG/HA-INS formulations, respectively. An analysis of the similarity of the release profiles was performed, and it was found that they are not similar (Table 1). The similarity coefficient (f2) was 48.23, while the difference coefficient (f1) was 34.63. It is assumed that the profiles are similar if the similarity coefficient (f2) is greater than 50 and the difference coefficient (f1) is less than 15 [35].

In order to explain the mechanism of insulin release from the developed hydrogels, comprehensive kinetic modelling of the obtained profiles was performed (Table 2). The release profiles were analysed based on selected mathematical models: zero-order model, first-order model, Higuchi model, Korsmeyer–Peppas model, Hixson–Crowell model, Peppas–Sahlin model, and Weibull model. It was found that insulin release is best described by the Peppas–Sahlin model (R^2^ > 0.99). This suggests that API (active pharmaceutical ingredient) release is mainly controlled by its diffusion from the hybrid system (kPS1 > kPS2), with limited relaxation of the polymer matrix. Hormone release is regulated by the hydration and structure of the polymer matrix [36]. A high fit of the INS release profiles to the Weibull model (R^2^ > 0.98) was also observed. The ranges of the shape parameter β were β HPMC/HA-INS = 0.701 and β ALG/HA-INS = 0.801, respectively. In the case of HPMC/HA-INS, Fickian diffusion dominates (β ≤ 0.75), while for ALG/HA-INS, the process proceeds according to a mixed mechanism—Fickian diffusion combined with case II transport (0.75 < β < 1).

**Figure 2:**
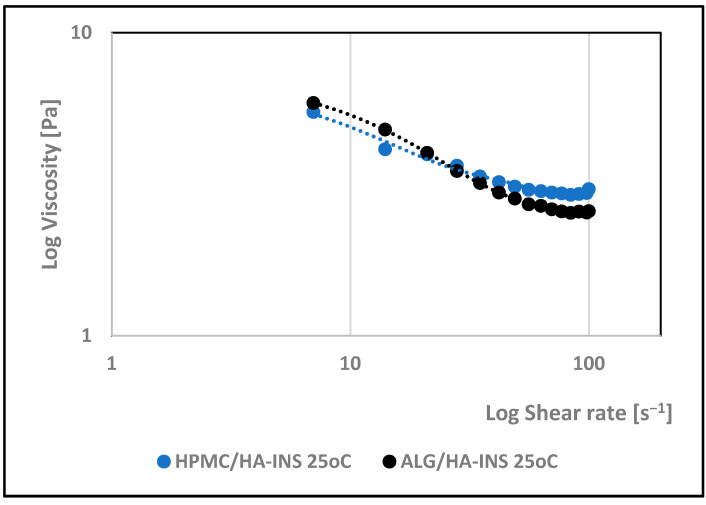
Effect of log shear rate on the log viscosity of HPMC/HA-INS and ALG/HA-INS hydrogels at 25 °C.

**Figure 3:**
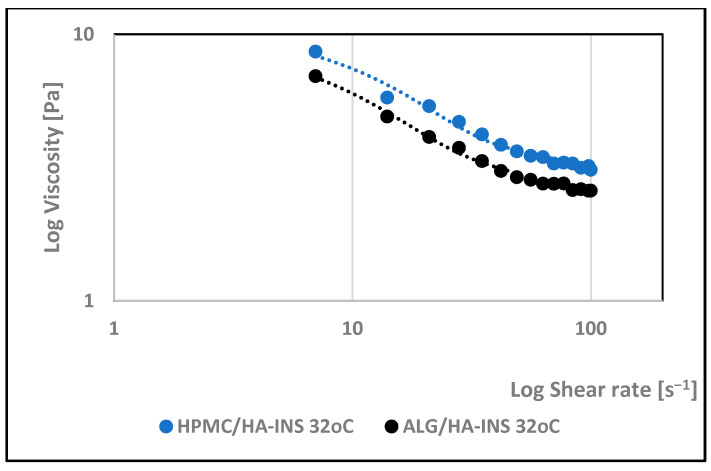
Effect of log shear rate on the log viscosity of HPMC/HA-INS and ALG/HA-INS hydrogels at 32 °C.

The effect of shear rate on the viscosity of the developed hydrogels was investigated. The rheological properties of the formulations were monitored at 25 °C and 32 °C. Analysis of the rheograms (Figure 2 and Figure 3) shows that the apparent viscosity decreases with increasing shear rate (7.0–100.0 s^−1^) and then stabilises, approaching the limit value. At shear rates > 40 s^−1^, the polymer chains show stronger orientation along the flow direction and arrange themselves into a more ordered structure. HPMC/HA-INS and ALG/HA-INS hydrogels behave as shear-thinning non-Newtonian fluids at both tested temperatures [37]. The analysed samples showed higher viscosity at 32 °C compared to the data obtained at 25 °C.

**Figure 4:**
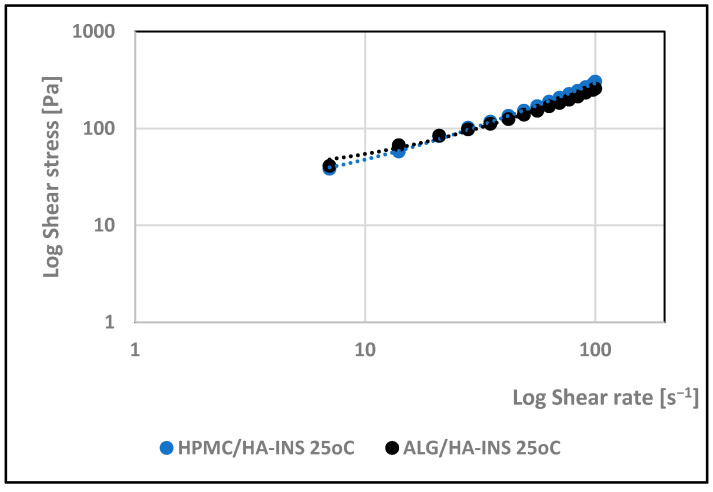
Log shear stress versus log shear rate for HPMC/HA-INS and ALG/HA-INS hydrogels at 25 °C.

**Figure 5:**
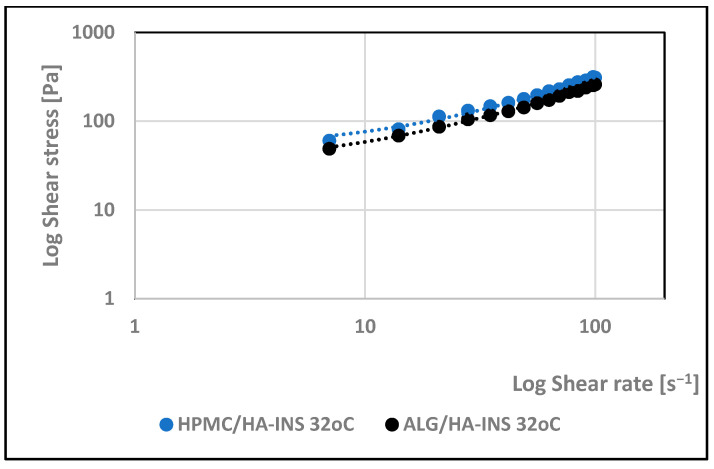
Log shear stress versus log shear rate for HPMC/HA-INS and ALG/HA-INS hydrogels at 32 °C.

Viscosity expresses the ratio of shear stress to shear rate (Figure 4 and Figure 5). Analysis of the flow curves showed an increase in shear stress with increasing shear rate for both formulations at the two temperatures analysed (25 °C and 32 °C). The experimental curves obtained were analysed using rheological models (Table 3). The coefficient of determination R^2^ was used as an indicator of the model’s fit to the analysed profile. The best fit was obtained for the Herschel–Bulkley model (pseudoplastic with yield stress) [38]. R^2^ is in the range of 0.997–0.998. The tested hydrogels have a yield stress that is lower at 25 °C (τ0HPMC/HA-INS = 16 Pa; τ0ALG/HA-INS = 14.4 Pa) than at 32 °C (τ0HPMC/HA-INS = 28.8 Pa; τ0ALG/HA-INS = 27.0 Pa). A value of *n* less than 1 indicates that the formulations at both temperatures exhibit shear thinning properties, which indicates ease of distribution on diseased tissue, high spreadability, and retention at the application site without leakage [39,40,41].

**Figure 6:**
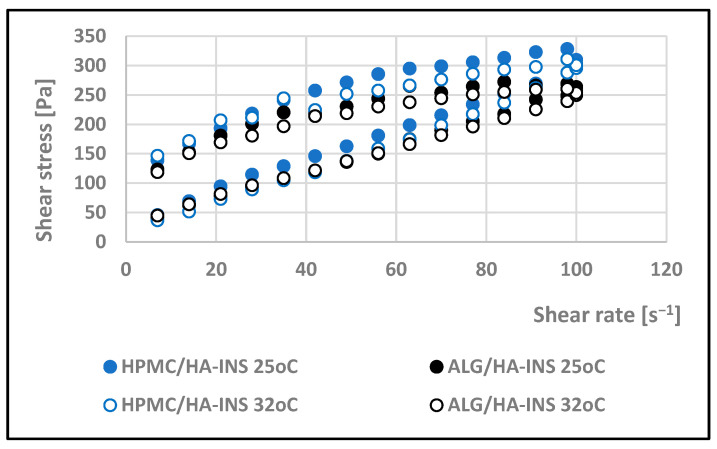
Hysteresis loops for HPMC/HA-INS and ALG/HA-INS hydrogels at 25 °C and 32 °C.

The thixotropic properties of the tested systems were determined using a hysteresis loop test. Viscosity was measured at increasing and then decreasing shear rates. The resulting hysteresis loops are shown in Figure 6. The surface area of the hysteresis loop reflects the amount of energy required to destroy the hydrogel matrix structure [42]. At 25 °C, the surface areas were 8237.511 Pa/s (HPMC/HA-INS) and 7328.551 Pa/s (ALG/HA-INS), respectively, while at 32 °C, the values were 8651.133 Pa/s (HPMC/HA-INS) and 6426.959 Pa/s (ALG/HA-INS). The developed hydrogels exhibit thixotropic properties, which will enable their application and even distribution on the skin. The restoration of the original structure of the hydrogel matrix will prevent the hydrogels from leaking from the packaging. The fastest restoration of the structure at 25 °C and 32 °C will be ensured by the ALG/HA-INS preparation.

**Figure 7:**
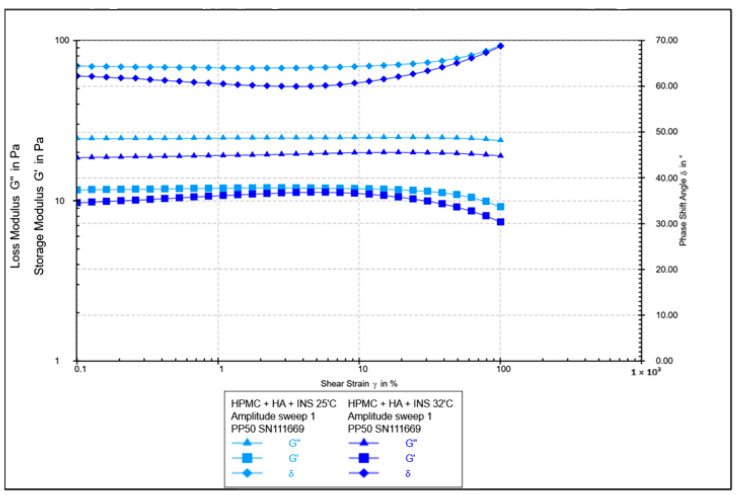
Results of the amplitude test as a function of shear strain for HPMC/HA-INS hydrogels at 25 °C and 32 °C.

**Figure 8:**
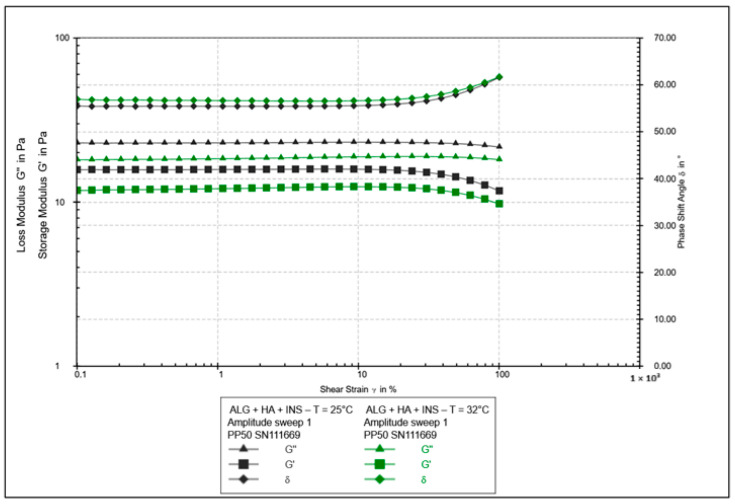
Results of the amplitude test as a function of shear strain for ALG/HA-INS hydrogels at 25 °C and 32 °C.

**Figure 9:**
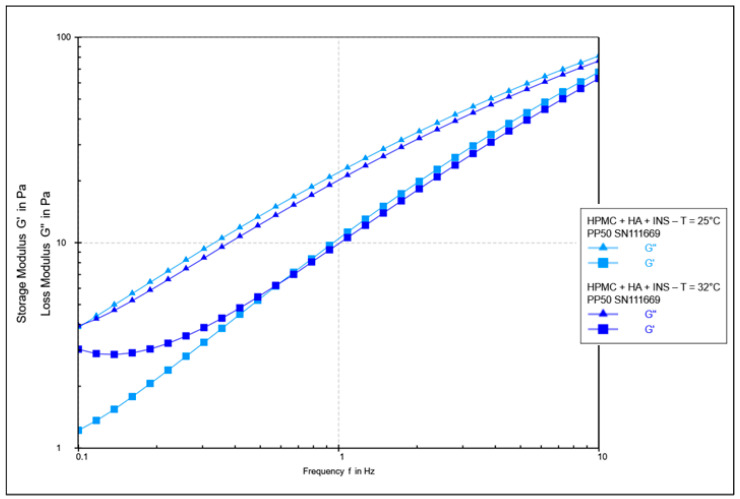
Sweeping of the HPMC/HA-INS sample frequency at 25 °C and 32 °C.

**Figure 10:**
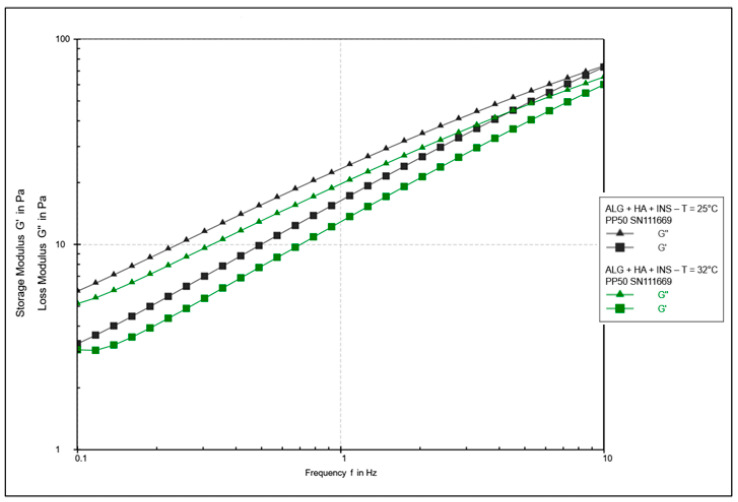
Sweeping the ALG/HA-INS sample frequency at temperatures of 25 °C and 32 °C.

The oscillatory rheology test assessed changes in the values of elasticity modules G′ and viscosity G″ (Figure 7, Figure 8, Figure 9 and Figure 10). In Figure 7 and Figure 8, the shear elasticity module G′ is constant, so the material is in the linear viscoelastic range, where the applied deformation does not lead to any damage to the structure. The increase in temperature caused a decrease in the elasticity and viscosity modules and an increase in the phase angle with increasing shear stresses (>45°). Hydrogels at 25 °C exhibit higher stiffness.

Figure 9 and Figure 10 show the elasticity and viscosity modulus curves during frequency sweeping. It was found that the G′ value is lower than the G″ value at both analysed temperatures, which indicates the predominance of viscous characteristics over viscoelastic characteristics. No transition between elastic and viscous behaviour (G′ = G″) was observed in the measured frequency range, suggesting that it probably occurs at higher frequencies. The G′ and G″ curves tend to converge with increasing frequency. It is suggested that polymer matrices take on a more solid form at higher frequencies [43]. Across the entire measurement range, the elastic modulus G′ was lower for the HPMC/HA-INS sample (vs. ALG/HA-INS) at both 25 °C and 32 °C. In turn, the viscosity modulus (G″) was lower for the ALG/HA-INS sample (vs. HPMC/HA-INS) at both temperatures. The increase in temperature led to a decrease in both the elasticity and viscosity moduli. The analysed hydrogels exhibit viscoelastic properties similar to those of liquids [44]. Some authors, analysing dispersions of alginate and cellulose derivatives, also observed the dominance of the viscosity modulus over the elasticity modulus in frequency sweep tests [23,45,46]. This is probably due to the dispersion of energy during the rearrangement of chains and bonds [46].

Analysis of texture profiles (TPA and CRT) allows the mechanical properties of the developed hydrogels to be determined. The height of the positive peaks on the TPA graphs describes the hardness properties of the formulation (the force required for compression). This value should be low to allow easy extrusion of the preparation from the container and optimal application. Adhesion (the area of the first negative peak) reflects the work required to overcome the attractive force between the probe (the surface of the materials) and the surface of the formulation. This parameter is often equated with mucoadhesion [47,48]. In addition, a correlation has been found between the bioadhesive properties of the preparation and its adhesion [49].

**Figure 11:**
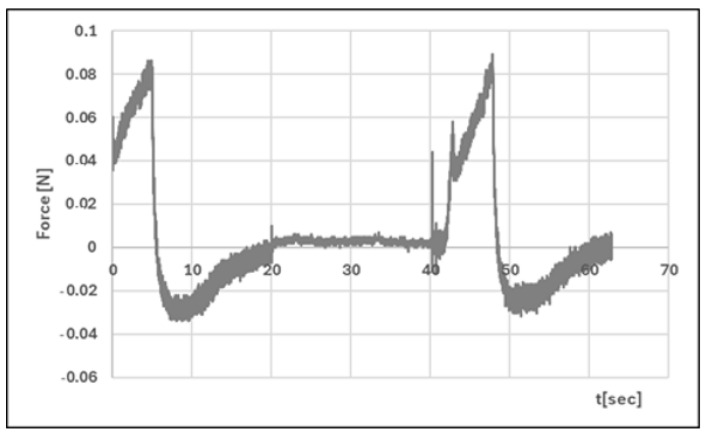
Texture profile analysis (TPA) of ALG/HA-INS.

**Figure 12:**
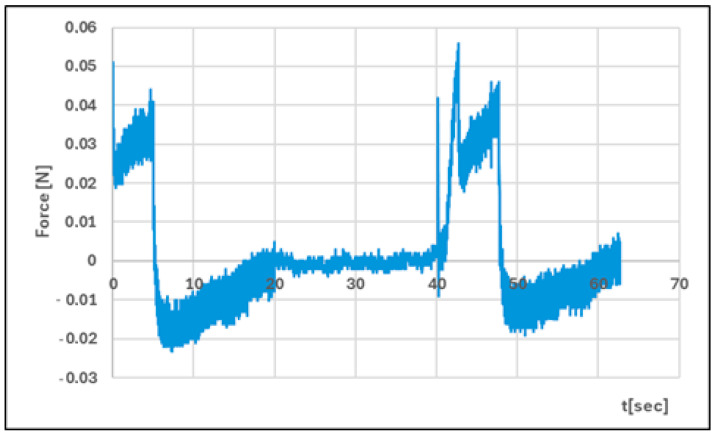
Texture profile analysis (TPA) of HPMC/HA-INS.

**Figure 13:**
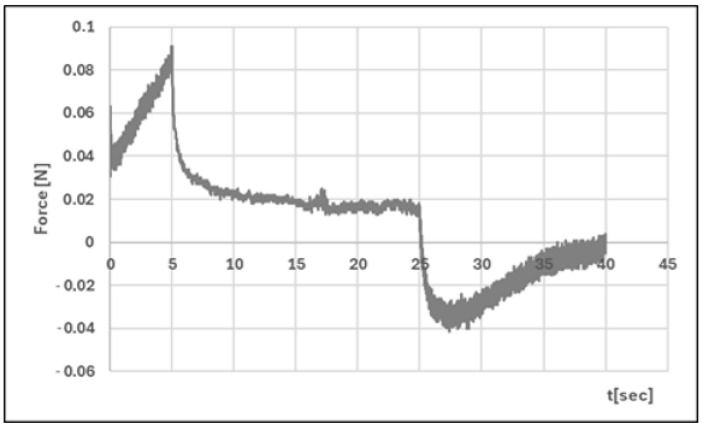
Penetration test (CRT) of ALG/HA-INS.

**Figure 14:**
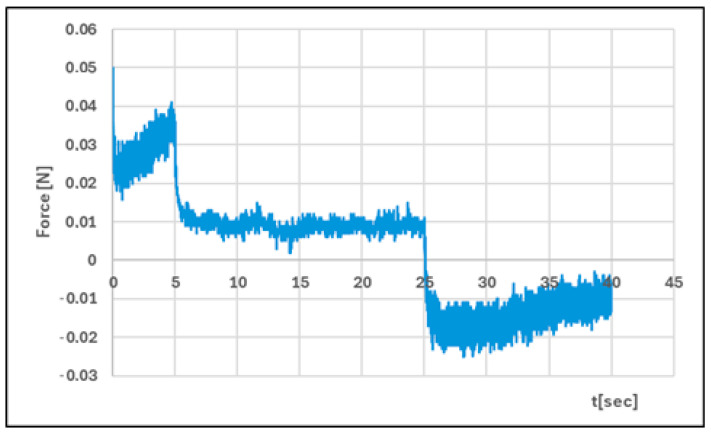
Penetration test (CRT) of HPMC/HA-INS.

The adhesive capacity of the hydrogel ensures that the drug remains at the site of application and maintains its clinical efficacy [50]. Cohesiveness (the ratio of the area under the second positive peak to the first positive peak) determines the structural recovery of the hydrogel after the compression stage. This parameter expresses the ability of the preparation to reversibly reduce its volume under load [39,51]. Table 4 and Figure 11, Figure 12, Figure 13 and Figure 14 present the results of the TPA and CRT tests. The ALG/HA-INS hydrogel was characterised by greater hardness (0.086 ± 0.02 N vs. HPMC/HA-INS: 0.051 ± 0.01 N *p* < 0.05). No statistically significant differences were found in the values of the parameters: cohesiveness, adhesiveness, and elasticity. The analysed hydrogels will ensure optimal adhesion and retention at the application site. The results obtained correspond to the data presented in the literature [23,52,53,54].

## 4. Discussion

The study proposed two hydrogel systems, alginate-hyaluronan (ALG/HA) and hydroxypropyl methylcellulose-hyaluronan (HPMC/HA), as insulin carriers for potential use on the skin. The polymers were selected based on their documented application properties in drug delivery [4,9,11,55,56] and their potential for clinical use. Designing a hydrogel carrier that enables effective delivery of API through the skin is a challenge. The stratum corneum, the outermost layer of the epidermis (the so-called barrier layer), hinders the diffusion of the drug through the skin [57]. In order for the process of insulin penetration through the skin to be effective, the formulations were supplemented with glycerol. This substance increases the penetration of API through the skin and has anti-inflammatory and moisturising properties. The structure of glycerol contains electronegative -OH groups, which can form hydrogen bonds with the -NH groups of ceramides (a component of the skin’s hydrolipid barrier). This process disrupts the integrity of the skin barrier and improves the diffusion of the API through the skin [39,58,59]. An additional advantage of the developed hydrogels is the introduction of insulin (Insulatard Penfil) into the formulation, which contains metacresol and phenol with antimicrobial properties. This reduces the risk of microbial contamination. The zinc chloride present in the pharmaceutical preparation may inhibit protease activity and affect insulin stability at the wound site [60].

Studies conducted on the pharmaceutical availability of insulin from HPMC/HA-INS and ALG/HA-INS hydrogels through the Strat-M^®^ membrane indicate that after 540 min of testing, 43% and 57% of the initial hormone dose was released, respectively. The release was prolonged, according to the Peppas–Sahlin kinetic model (R^2^ > 0.99), and was regulated by hydration and matrix structure (kPS1 > kPS2). The lower percentage of insulin released from HPMC/HA-INS hydrogel vs. ALG/HA-INS hydrogel is primarily due to the viscosity of the analysed systems. For HPMC/HA-INS at 32 °C ± 1 °C, the measured viscosity values at selected shear rates were 30 [s^−1^] 2.841 ± 0.9088 [Pa·s]; 50 [s^−1^]: 2.132 ± 0.6714 [Pa·s]; 100 [s^−1^]: 1.619 ± 0.4982 [Pa·s], while for ALG/HA-INS, the values were 30 [s^−1^]: 2.704 ± 0.8618 [Pa·s]; 50 [s^−1^]: 2.087 ± 0.7376 [Pa·s]; 100 [s^−1^]: 1.480 ± 0.4589 [Pa·s] (Figure 3). The smaller hysteresis loop area (*p* = 8651.133 Pa/s—HPMC/HA-INS) and *p* = 6426.959 Pa/s—ALG/HA-INS) also contributed to higher API availability, which was confirmed in other studies [61,62,63]. This suggests a stronger bond between the HPMC/HA matrix system and insulin. The osmotic pressure of the hydrogel (HPMC/HA-INS: 448 mOsm/L, ALG/HA-INS: 974 mOsm/L) has an additional effect on API release. During the bioavailability study, the hydrogels swelled and increased in volume, driven by the pressure difference between the formulation and the surrounding PBS model fluid. This process resulted in the loosening of the polymer matrix structure and the release of the API. This suggests that the higher osmotic pressure of the ALG/HA-INS hydrogel (974 mOsm/L) may also have contributed to the higher amount of insulin released [63].

The therapeutic efficacy of hydrogels is closely related to their rheological and textural properties. HPMC/HA-INS and ALG/HA-INS hydrogels exhibited non-Newtonian shear-thinning properties at both tested temperatures. This indicates that the formulation will spread easily on the skin and be optimally dispensed from the unit packaging. Oscillatory tests confirmed that the consistency of hydrogels is more viscous (“viscoelastic liquids”), with a tendency for G′ < G″. A similar relationship for the ALG/HA system was observed by Antich et al. [64]. Hydrogels behaved as viscous materials over a wide frequency range. Cuomo et al. [65] suggest that the structure of alginate-based hydrogels depends on the amount of Ca^2+^ ions contained in the matrix. As the concentration of calcium ions increases, G′ increases and, consequently, the stiffness of the hydrogel increases. Our previous preformulation studies of chitosan (CS)-based hydrogels with cellulose derivatives (CS/MC-INS, CS/HEC-INS, CS/HPMC-INS) also confirmed the dominant “liquid-like” structure for these systems [23]. This suggests that hydrogels will adapt to irregularly shaped wounds. An additional sterile dressing may support local retention of the formulation in the affected area. The developed hydrogels also exhibit favourable textural properties. No statistically significant differences were found in the values of the parameters: cohesiveness, adhesiveness, and elasticity. The hardness parameters were <1 and within the acceptable range, with the ALG/HA-INS hydrogel exhibiting greater hardness.

Compared to previous studies on the formulation of hybrid insulin carriers [23], the amount of hormone released was satisfactory. From the CS/HPMC (chitosan/hydroxypropyl methylcellulose) hydrogel, 49% of the INS dose was released after 6.5 h. The remaining CS/HEC (chitosan/hydroxyethylcellulose) and CS/MC (chitosan/methylcellulose) carriers released 42.5% (after 7 h) and 39.8% (after 7 h) of insulin, respectively [23]. In this study, after 9 h, 43% and 57% of the initial hormone dose were released from the HPMC/HA-INS and ALG/HA-INS formulations, respectively. It should also be emphasised that the combination of natural and synthetic polymers in a single matrix allows for the creation of a preparation with high biocompatibility and optimal mechanical properties, in line with application requirements. The appropriate selection of hybrid hydrogel components allows for the regulation of rheological parameters and API release kinetics.

## 5. Conclusions

The developed HPMC/HA-INS and ALG/HA-INS hydrogels provide a balance between rheological properties, texture, and optimal application to the skin. Insulin was released in a sustained manner, eliminating the need for frequent application of the hydrogel. It seems advisable to conduct cytotoxicity and biocompatibility tests on the developed hydrogel formulations in order to comprehensively assess their safety.

## Figures and Tables

**Table 1:** Comparison of the release profiles of HPMC/HA-INS and ALG/HA-INS hydrogels.

Formula Code	Equation	Result	Interpretation
f1 HPMC/HA-INS vs. ALG/HA-INS	f1 = $\left[\frac{\sum \left|R_{t}-T_{t}\right|}{\sum R_{t}}\right]\cdot 100$	34.63	Dissimilar
f2 HPMC/HA-INS vs. ALG/HA-INS	f2 = 50 log$\left\{{\left[1+\frac{1}{n}\sum {\left(R_{t}-T_{t}\right)}^{2}\right]}^{-0.5}\cdot 100\right\}$	48.23	Dissimilar

Symbols: f_1_, difference factor; f_2_, similarity factor; *n*, number of time points; Rt, reference sample at time t; Tt, test sample at time t.

**Table 2:** Mathematical models describing insulin release profiles from hydrogels.

Model	Equation	Hydrogel HPMC/HA-INS (Parameters, R^2^ adj, AIC, MSC)	Hydrogel ALG/HA- INS (Parameters, R^2^ adj, AIC, MSC)
Zero-order	F = k_0_ t	k_0_ = 0.099 R^2^ _adj_ = 0.8371 AIC = 139.1119 MSC = 1.5305	k_0_ = 0.139 R^2^ _adj_ = 0.8458 AIC = 143.5498 MSC = 1.5959
First-order	F = 1−e^−k^_1_^t^	k_1_ = 0.001 R^2^ _adj_ = 0.9302 AIC = 121.3200 MSC = 2.3778	k_1_ = 0.002 R^2^ _adj_ = 0.9592 AIC = 116.9775 MSC = 2.9245
Higuchi	F= k_H_ t^0.5^	k_H_ = 1.927 R^2^ _adj_ = 0.9735 AIC = 100.9586 MSC=3.3474	k_H_ = 2.616 R^2^ _adj_ = 0.9503 AIC = 120.9035 MSC = 2.7282
Korsmeyer–Peppas	F = k_KP_ t^n^	k_KP_ = 1.181 *n* = 0.584 R^2^ _adj_ = 0.9825 AIC = 93.2225 MSC = 3.7158	k_KP_ = 1.381 *n* = 0.611 R^2^ _adj_ = 0.9644 AIC = 115.1723 MSC = 3.0148
Hixson–Crowell	F = 1−(1−k_HC_ t)^3^]	k_HC_ = 0.001 R^2^ _adj_ = 0.9195 AIC = 138.1944 MSC = 2.2501	k_HC_ = 0.001 R^2^ _adj_ = 0.9330 AIC = 126.8927 MSC = 2.4288
Peppas–Sahlin	F = k_PS1_ t^m^ + k_PS2_ t^2m^	k_PS1_ = 0.308 k_PS2_ = −0.001 m = 0.890 R^2^ _adj_ = 0.9993 AIC = 27.3617 MSC = 6.8520	k_PS1_ = 0.244 k_PS2_ = 0.000 m = 0.998 R^2^ _adj_ = 0.9967 AIC = 68.2465 MSC = 5.3611
Weibull	F = 100 (1−e^−(t^β)/α^)	α = 133.388 β = 0.701 R^2^ _adj_ = 0.9894 AIC = 82.6533 MSC = 4.2190	α = 155.449 β = 0.801 R^2^ _adj_ = 0.9801 AIC = 103.4961 MSC = 3.5986

Symbols: F, cumulative quantity of drug released at time *t*; k_0_, reaction rate coefficient; k_1_, rate constant; k_H_, dissolution constant; k_HC_, Hixson–Crowell release constant; k_KP_, constant depicting the experimental parameters based on geometry and dosage forms; k_PS1_, Peppas–Sahlin release constant (constant for Fickian diffusion); k_PS2_, constant for Case II relaxational mechanism; m, diffusion exponent; *n*, release exponent (*n* ≤ 0.45 Fickian diffusion, 0.45 < *n* < 0.89 non-Fickian transport, *n* = 0.89 case II (relaxation) transport, *n* > 0.89 super case II transport mechanism); R^2^ adj, Adjusted R-squared; t, time; α, scale parameter; β, shape parameter. AIC, Akaike Information Criterion: AIC = *n*ln(WSS) + 2*p* (where *n*, number of data points; WSS, weighted sum of squares; *p*, number of parameters in the model). MSC, Model Selection Criterion: MSC = ln$\left(\frac{\sum_{i=1}^{n}w_{i}\cdot {\left(y_{i\_obs}-{\bar{y}}_{\_obs}\right)}^{2}}{\sum_{i=1}^{n}w_{i}\cdot {\left(y_{i\_obs}-y_{i\_pre}\right)}^{2}}\right)\;-\;\frac{2p}{n}$ where w_i_, weighting factor; y_i__obs, i-th observed *y* value; y_i_pre_, i-th predicted *y* value; $\overset{-}{y}$__obs_, mean of all observed y-data points; *p*, number of parameters in the model; *n*, number of data points).

**Table 3:** The results obtained from the mathematical modeling of rheogram.

Hydrogel	Herschel–Bulkley				Ostwald–de Waele			Bingham		Casson	
	τ0	*n*	K	R^2^	*n*	K	R^2^	τ0	R^2^	τ0	R^2^
	25 °C										
HPMC/HA-INS	16.000	0.94	3.60	0.998	0.780	7.66	0.994	20.533	0.997	4.309	0.997
ALG/HA-INS	14.400	0.794	5.91	0.997	0.674	10.7	0.992	32.627	0.995	10.236	0.996
	32 °C										
HPMC/HA-INS	28.800	0.822	6.34	0.997	0.633	16.1	0.991	49.837	0.996	17.353	0.996
ALG/HA-INS	27.00	0.873	4.06	0.998	0.639	12.7	0.988	37.722	0.997	12.920	0.997

Symbols: τ0, the Yield stress [Pa]; K, the consistency index [Pa*sn]; *n*, the flow behavior index; R^2^, regression coefficient.

**Table 4:** Texture parameters of hydrogel (Mean ± SD, *n* = 3, T = 25 °C ± 0.1 °C).

Parameter	Hydrogel HPMC/HA-INS (Mean ± SD)	Hydrogel ALG/HA-INS (Mean ± SD)	*p*
Relaxation [%]	86.9 ± 0.88	81.8 ± 0.97	*p* < 0.01
Hardness 1 [N]	0.051 ± 0.01	0.086 ± 0.02	*p* < 0.05
Hardness 2 [N]	0.056 ± 0.01	0.089 ± 0.01	*p* < 0.05
Cohesiveness [-]	1.088 ± 0.08	0.997 ± 0.20	NS
Adhesiveness [mJ]	0.2 ± 0.05	0.2 ± 0.10	NS
Elasticity [-]	1.016 ± 0.05	1.141 ± 0.11	NS

## Data Availability

The original contributions presented in this study are included in the article. Further inquiries can be directed to the corresponding author.

## References

[1] Sopata M., Jawień A., Mrozikiewicz-Rakowska B., Augusewicz Z., Bakowska M., Samson I., Gabriel M., Grzela T., Karpiński T., Kuberka I. (2020). Wytyczne postępowania miejscowego w ranach niezakażonych, zagrożonych infekcją oraz zakażonych—Przegląd dostępnych substancji przeciwdrobnoustrojowych stosowanych w leczeniu ran. Zalecenia Polskiego Towarzystwa Leczenia Ran. Leczenie Ran.

[2] (2022). European Pharmacopoeia.

[3] Kumar A., Sah D.K. (2023). A calcium and zinc composite alginate hydrogel for pre-hospital hemostasis and wound care. Carbohydr. Polym..

[4] Catanzano O., Esposito V.D., Acierno S., Ambrosio M.R., Caro C.D., Avagliano C., Russo P., Russo R., Miro A., Ungaro F. (2015). Alginate—Hyaluronan composite hydrogels accelerate wound healing process. Carbohydr. Polym..

[5] Abka-khajouei R., Tounsi L., Shahabi N., Patel A.K., Abdelkafi S., Michaud P. (2022). Structures, Properties and Applications of Alginates. Mar. Drugs.

[6] Slaughter B.V., Khurshid S.S., Fisher O.Z., Khademhosseini A., Peppas N.A. (2009). Hydrogels in Regenerative Medicine. Adv. Mater..

[7] Toole B.P. (2004). Hyaluronan: From Extracellular Glue to Pericellular Cue. Nat. Rev. Cancer.

[8] Petrey A.C., De La Motte C.A. (2014). Hyaluronan, a crucial regulator of inflammation. Front Immunol..

[9] Zarei N., Hassanzadeh-Tabrizi S. (2023). Alginate/hyaluronic acid-based systems as a new generation of wound dressings: A review. Int. J. Biol. Macromol..

[10] Voigt J., Driver V.R. (2012). Hyaluronic acid derivatives and their healing effect on burns, epithelial surgical wounds, and chronic wounds: A systematic review and meta-analysis of randomized controlled trials. Wound Repair Regen..

[11] Tudoroiu E.-E., Dinu-Pîrvu C.-E., Albu Kaya M.G., Popa L., Anuța V., Prisada R.M., Ghica M.V. (2021). An Overview of Cellulose Derivatives-Based Dressings for Wound-Healing Management. Pharmaceuticals.

[12] Al-Bazzaz F.Y., Ismail S.T. (2024). Topical HPMC/Carbopol 934 gel for wound healing: Formulation and in-vivo evaluation. Pharmakeftiki.

[13] Ostróżka-Cieślik A., Przybyła M., Wójcik W., Birówka K., Majczyna M., Dolińska B. (2023). Review of Research in Developing Hydrogels with Insulin to Promote Wound Healing. Med. Sci. Forum.

[14] Przybyła M., Dolińska B., Ostróżka-Cieślik A. (2023). Research Progress on Insulin Dressings to Promote Wound Healing. Eng. Proc..

[15] Przybyła M., Dolińska B., Ostróżka-Cieślik A. (2024). Progress of knowledge in the development of chitosan formulations with insulin to promote skin wound healing. Prog. Chem. Appl. Chitin Deriv..

[16] Vatankhah N., Jahangiri Y., Landry G.J., Moneta G.L., Azarbal A.F. (2017). Effect of systemic insulin treatment on diabetic wound healing. Wound Repair Regen..

[17] Zhang X.J., Chinkes D.L., Wolf S.E., Wolfe R.R. (1999). Insulin but not growth hormone stimulates protein anabolism in skin wound and muscle. Am. J. Physiol..

[18] Rezvani O., Shabbak E., Aslani A., Bidar R., Jafari M., Safarnezhad S. (2009). A randomized, double-blind, placebo-controlled trial to determine the effects of topical insulin on wound healing. Ostomy/Wound Manag..

[19] Wilson J.M., Baines R., Babu E.D., Kelley C.J. (2008). A role for topical insulin in the management problematic surgical wounds. Ann. R. Coll. Surg. Engl..

[20] Chen X., Zhang X., Liu Y. (2012). Effect of topical insulin application on wound neutrophil function. Wounds.

[21] Hrynyk M., Neufeld R.J. (2014). Insulin and wound healing. Burns.

[22] Mumuni A.M., Calister E.U., Aminu N., Franklin C.K., Musiliu Oluseun A., Usman M., Abdulmumuni B., James Y.O., Ofokansi C.K., Anthony A.A. (2020). Mucin-Grafted Polyethylene Glycol Microparticles Enable Oral Insulin Delivery for Improving Diabetic Treatment. Appl. Sci..

[23] Ostróżka-Cieślik A., Strasser C., Dolińska B. (2024). Insulin-Loaded Chitosan–Cellulose-Derivative Hydrogels: In Vitro Permeation of Hormone through Strat-M^®^ Membrane and Rheological and Textural Analysis. Polymers.

[24] Muselík J., Komersová A., Kubová K., Matzick K., Skalická B. (2021). A Critical Overview of FDA and EMA Statistical Methods to Compare In Vitro Drug Dissolution Profiles of Pharmaceutical Products. Pharmaceutics.

[25] Zhang Y., Huo M., Zhou J., Zou A., Li W., Yao C., Xie S. (2010). DDSolver: An Add-In Program for Modeling and Comparison of Drug Dissolution Profiles. AAPS J..

[26] Ostróżka-Cieślik A., Wilczyński S., Dolińska B. (2023). Hydrogel Formulations for Topical Insulin Application: Preparation, Characterization and In Vitro Permeation across the Strat-M^®^ Membrane. Polymers.

[27] Ostróżka-Cieślik A., Maciążek-Jurczyk M., Pożycka J., Dolińska B. (2021). Pre-Formulation Studies: Physicochemical Characteristics and In Vitro Release Kinetics of Insulin from Selected Hydrogels. Pharmaceutics.

[28] Hurler J., Engesland A., Poorahmary Kermany B., Škalko-Basnet N. (2012). Improved texture analysis for hydrogel characterization: Gel cohesiveness, adhesiveness, and hardness. J. Appl. Polym. Sci..

[29] Lee D., Zhang H., Ryu S., Mondal M.I.H. (2018). Elastic modulus measurement of hydrogels. Cellulose-Based Superabsorbent Hydrogels.

[30] Yılmaz Usta D., Teksin Z.S., Tugcu-Demiroz F. (2024). Evaluation of Emulgel and Nanostructured Lipid Carrier-Based Gel Formulations for Transdermal Administration of Ibuprofen: Characterization, Mechanical Properties, and Ex-Vivo Skin Permeation. AAPS PharmSciTech.

[31] Rafiee A., Mozafari N., Fekri N., Memarpour M., Azadi A. (2024). Preparation and Characterization of a Nanohydroxyapatite and Sodium Fluoride Loaded Chitosan-Based in Situ Forming Gel for Enamel Biomineralization. Heliyon.

[32] Ostróżka-Cieślik A. (2022). The Potential of Pharmaceutical Hydrogels in the Formulation of Topical Administration Hormone Drugs. Polymers.

[33] Snetkov P., Zakharova K., Morozkina S., Olekhnovich R., Uspenskaya M. (2020). Hyaluronic Acid: The Influence of Molecular Weight on Structural, Physical, Physico-Chemical, and Degradable Properties of Biopolymer. Polymers.

[34] Hering A., Cal K., Kowalczyk M., Kastsevich A., Ivashchanka Y., Ochocka J.R., Stefanowicz-Hajduk J. (2025). Bufadienolide Penetration Through the Skin Membrane and Antiaging Properties of *Kalanchoe* spp. Juices in Dermal Applications. Molecules.

[35] Khadka A., Giri B.R., Baral R., Shakya S., Shrestha A.K. (2025). Formulation and In Vitro Characterization of Cellulose-Based Propranolol Hydrochloride Sustained Release Matrix Tablets. BioChem.

[36] Kshirsagar M.M., Chatale B.C., Dyawanapelly S., Vora L.K., Amin P.D. (2025). Continuous Processing Strategies for Amorphous Solid Dispersions of Itraconazole: Impact of Polymer Selection and Manufacturing Techniques. Pharmaceutics.

[37] Kokol V., Pottathara Y.B., Mihelčič M., Perše L.S. (2021). Rheological properties of gelatine hydrogels affected by flow-and horizontally-induced cooling rates during 3D cryo-printing. Colloids Surf. A Physicochem. Eng. Asp..

[38] Angar N.E., Aliouche D. (2016). Rheological behavior and reversible swelling of pH sensitive poly(acrylamide-*co*-itaconic acid) hydrogels. Polym. Sci. Ser. A.

[39] Kadian V., Rao R. (2024). Enhancing anti-inflammatory effect of brucine nanohydrogel using rosemary oil: A promising strategy for dermal delivery in arthritic inflammation. 3 Biotech.

[40] Dantas M.G.B., Reis S.A.G.B., Damasceno C.M.D., Rolim L.A., Rolim-Neto P.J., Carvalho F.O., Quintans-Junior L.J., Almeida J.R. (2016). Development and evaluation of stability of a gel formulation containing the monoterpene borneol. Sci. World J..

[41] Garg N.K., Sharma G., Singh B., Nirbhavane P., Tyagi R.K., Shukla R., Katare O.P. (2017). Quality by Design (QbD)-enabled development of aceclofenac loaded-nano structured lipid carriers (NLCs): An improved dermatokinetic profile for inflammatory disorder(s). Int. J. Pharm..

[42] Huang N., Sun J., Liu J., Lv K., Deng X., Zhang T., Sun Y., Yan H., Hou D. (2025). Enhancing Rheology and Wettability of Drilling Fluids at Ultra-Low Temperatures Using a Novel Amide Material. Gels.

[43] Steffe J.F. (1996). Rheological Methods in Food Process Engineering.

[44] Budai L., Budai M., Fülöpné Pápay Z.E., Vilimi Z., Antal I. (2023). Rheological Considerations of Pharmaceutical Formulations: Focus on Viscoelasticity. Gels.

[45] Eskens O., Villani G., Amin S. (2021). Rheological Investigation of Thermoresponsive Alginate-Methylcellulose Gels for Epidermal Growth Factor Formulation. Cosmetics.

[46] Liparoti S., Speranza V., Marra F. (2021). Alginate hydrogel: The influence of the hardening on the rheological behaviour. J. Mech. Behav. Biomed. Mater..

[47] Özgüney I., Kardhiqi A. (2014). Properties of bioadhesive ketoprofen liquid suppositories: Preparation, determination of gelation temperature, viscosity studies and evaluation of mechanical properties using texture analyzer by 4 × 4 factorial design. Pharm. Dev. Technol..

[48] Baloglu E., Karavana S.Y., Senyigit Z.A., Hilmioglu-Polat S., Metin D.Y., Zekioglu O., Guneri T., Jones D.S. (2011). In-situ gel formulations of econazole nitrate: Preparation and in-vitro and in-vivo evaluation. J. Pharm. Pharmacol..

[49] Jones D.S., Woolfson A.D., Brown A.F. (1997). Textural, viscoelastic and mucoadhesive properties of pharmaceutical gels composed of cellulose polymers. Int. J. Pharm..

[50] Woolfson A.D., McCafferty D.F., Gorman S.P., McCarron P.A., Price J.H. (1992). Design of an Apparatus Incorporating a Linear Variable Differential Transformer for the Measurement of Type III Bioadhesion to Cervical Tissue. Int. J. Pharm..

[51] Rani P., Verma V., Kumar S., Bhatia M. (2024). Isolation, characterization and evaluation of pineapple crown waste nanofiber gel entrapping ampicillin in topical bacterial infections. Iran. Polym. J..

[52] Bruschi M.L., Jones D.S., Panzeri H., Gremião M.P., De Freitas O., Lara E.H. (2007). Semisolid systems containing propolis for the treatment of periodontal disease: In vitro release kinetics, syringeability, rheological, textural, and mucoadhesive properties. J. Pharm. Sci..

[53] Jones D.S., Woolfson A.D., Brown A.F. (1997). Textural analysis and flow rheometry of novel, bioadhesive antimicrobial oral gels. Pharm. Res..

[54] Vitorino C., Alves L., Antunes F.E., Sousa J.J., Pais A.A.C.C. (2013). Design of a Dual Nanostructured Lipid Carrier Formulation Based on Physicochemical, Rheological, and Mechanical Properties. J. Nanopart. Res..

[55] Sudhakar K., Ji S.m., Kummara M.R., Han S.S. (2022). Recent Progress on Hyaluronan-Based Products for Wound Healing Applications. Pharmaceutics.

[56] Ishfaq B., Khan I.U., Khalid S.H., Asghar S. (2023). Design and evaluation of sodium alginate-based hydrogel dressings containing Betula utilis extract for cutaneous wound healing. Front. Bioeng. Biotechnol..

[57] Mishra R., Jain N., Kaul S., Nagaich U. (2023). Central composite design-based optimization, fabrication, and pharmacodynamic assessment of sulfasalazine-loaded lipoidal nanoparticle-based hydrogel for the management of rheumatoid arthritis. Drug Deliv. Transl. Res..

[58] Fluhr J.W., Darlenski R., Surber C. (2008). Glycerol and the skin: Holistic approach to its origin and functions. Br. J. Dermatol..

[59] Ruan H., Shen L., Hou X., Li J., Guo T., Zhu C., Feng N., Zhang Y. (2023). Phytosterol-Mediated Glycerosomes Combined with Peppermint Oil Enhance Transdermal Delivery of Lappaconitine by Modulating the Lipid Composition of the Stratum Corneum. Drug Deliv. Transl. Res..

[60] Maret W. (2013). Inhibitory zinc sites in enzymes. Biometals.

[61] Tuğcu-Demiröz F. (2017). Vaginal delivery of benzydamine hydrochloride through liposomes dispersed in mucoadhesive gels. Chem. Pharm. Bull..

[62] Sanad W.G., Bader Q.A., Mahdi F.M.S., Kabbani F. (2023). Formulation and in Vitro Evaluation of Moxifloxacin-Lidocaine Base as A Topical Hydrogel Dressing. J. Nat. Sci. Biol. Med..

[63] Kulkarni R.V., Biswanath D.S. (2018). Electrically responsive smart hydrogels in drug delivery: A review. J. Appl. Biomater. Biomech..

[64] Antich C., de Vicente J., Jiménez G., Chocarro C., Carrillo E., Montañez E., Gálvez-Martín P., Marchal J.A. (2020). Bio-inspired hydrogel composed of hyaluronic acid and alginate as a potential bioink for 3D bioprinting of articular cartilage engineering constructs. Acta Biomater..

[65] Cuomo F., Cofelice M., Lopez F. (2019). Rheological Characterization of Hydrogels from Alginate-Based Nanodispersion. Polymers.

